# Social incentive factors in interventions promoting sustainable behaviors: A meta-analysis

**DOI:** 10.1371/journal.pone.0260932

**Published:** 2021-12-08

**Authors:** Phu Nguyen-Van, Anne Stenger, Tuyen Tiet

**Affiliations:** 1 EconomiX, CNRS, UPL, University of Paris Nanterre, Nanterre, France; 2 TIMAS, Thang Long University, Hanoi, Vietnam; 3 BETA, INRAE & University of Strasbourg, Strasbourg, France; 4 University of Management and Technology, Hanoi, Vietnam; Universitat de Valencia, SPAIN

## Abstract

Based on a meta-analysis, this paper highlights the strength and relevance of several social incentive factors concerning pro-environmental behaviors, including social influence, network factors (like network size, network connection and leadership), trust in others, and trust in institutions. Firstly, our results suggest that social influence is necessary for the emergence of pro-environmental behaviors. More specifically, an internal social influence (i.e., motivating people to change their perceptions and attitudes) is essential to promote pro-environmental behaviors. Secondly, network connection encourages pro-environmental behaviors, meaning that the effectiveness of a conservation policy can be improved if connections among individuals are increased. Finally, trust in institutions can dictate individual behaviors to shape policy design and generate desired policy outcomes.

## Introduction

It has been highlighted in the literature that individuals could be incentivized to mitigate environmental issues (e.g., climate change, biodiversity conservation, etc.) via using monetary incentives. As an example, monetary incentives have been successfully implemented to motivate people to protect their living environment, e.g., providing payments based on the quantity of recycled waste or the amount of electricity reduced [1–3]. However, the effectiveness of monetary policies is questionable. Firstly, they are costly to implement [4]. For example, the Pigovian tax or cap-and-trade emission requires relatively high administrative and monitoring costs to be successfully implemented. Secondly, the effect of monetary policies is not always sustainable in the long run [5, 6]. Several studies have shown that environmental conservation programs cannot be easily achieved if they fail to motivate people in terms of environmental sustainability: Will people continue to conserve energy if they know that they will not receive any more payments for their efforts in the future [7, 8]? Thus, the crowding-out effect of an environmental policy is also essential and needs to be taken into account [9, 10]. Thirdly, people’s motives can also be good drivers of pro-environmental behaviors [11]. While policymakers mainly focus on how to effectively use monetary incentives to encourage individuals or industries to protect the environment, social incentives (i.e., non-monetary incentives) are also useful tools to mitigate individuals’ negative impacts on the environment [11, 12].

Several studies have indicated that people engage in pro-environmental (pro-social) behaviors because of individual social incentives, such as social norms or intrinsic/extrinsic motivations, namely “social influence” [13, 14]. Social influence refers to how individuals alter their attitudes and behaviors in response to the demands of their social environment (e.g., an expectation of others, conformity or altruism, etc.) [15–17]. For instance, providing energy consumption feedback or environmental messages is an effective way to encourage households’ energy conservation [7]. In this case, if an individual consumes less electricity while others do not, he or she would gain not only a benefit from saving energy but an image reward by comparison with his or her neighbors as well (e.g., the best in the neighborhood) [18, 19].

Some of the existing literature also qualifies social influence as internal influences (e.g., altruism, intrinsic motivation or other-regarding preferences) and external influences (e.g., social norms or extrinsic motivation) [20, 21]. According to the theory of planned behavior (TPB), *external influence factors* are defined as social pressure or social norms that affect individual intentions to perform a target behavior. In contrast, the existing literature has suggested that attitude and personal norms are internal motives that could explain pro-environmental behaviors through intrinsic motivations [22–24]. For instance, it is essential to alter or strengthen citizens’ beliefs and perceptions about environmental protection to motivate them to take actions to mitigate climate change.

However, focusing on individual social incentives when addressing environmental issues may raise several problems. As for monetary contribution, for example, an individual who takes actions to alleviate his or her sense of obligation to help improve environmental quality may not take any further actions when he or she realizes that others do not cooperate (i.e., a single action bias). Furthermore, for most environmental issues (e.g., biodiversity, deforestation, energy, etc.), it is important to have many individuals, most often within the same area, adhering to a conservation program in order to reach a necessary threshold (i.e., the proportion of individuals in the network) above which a positive program effect can arise [25]. Thus, in addition to social influence, network factors and individual trust can also be used to promote “collective pro-environmental behaviors”, which are behaviors taken together by a group of individuals and including society as a whole (i.e., collective actions) to achieve an environmental target [25, 26].

In today’s world of social relationships, everyone is linked to a social network (e.g., the limited network of family, friends, relatives, neighbors, co-workers and even acquaintances). Since individuals are linked to each other, other individual behaviors could be an important factor that can be used to motivate a person to perform a specific action [27]. For example, people are more likely to adopt behaviors that are approved by others in order to cultivate or maintain close social relationships with others [17]. Some studies have shown that people who have been motivated by strong social influences may require pressure from their network to live up to their intentions [28]. Different network structures (characterized by different network size, network connection or degree of connection, and leadership) may have different impacts on individual contributions to a collective good [25]. In their study, the authors showed that a volunteer who is centrally located in a sparse network (i.e., network with a low degree of connection) has a more significant impact on others’ contributions than the one who is centrally located in a dense and less centralized network [25].

Besides network factors, individual trust is an important concept since trust is applicable to the relationship between people [29, 30]. Higher levels of trust (social and/or institutional) help ensure stronger social connections, which could indeed strengthen individual pro-environmental actions. Pro-environmental actions cannot be sustained if there is neither trust among individuals (i.e., trust in others) nor trust toward the institutions (e.g., government or leaders). Therefore, policymakers should also pay attention to social factors, such as network factors and trust, to motivate individual as well as collective actions to achieve an environmental target [31].

Several studies have provided descriptive reviews of this area of research, focusing on how information strategies influence energy conservation [32, 33], how social influence approaches can be used to encourage resource conservation [13, 34], presenting comparative studies of household energy conservation [35], analyzing determinants and outcomes of belief in climate change [36], testing behavioral inventions on climate change mitigation [37], and examining the evidence of spillover in pro-environmental behavior [38]. Although numerous studies have been conducted to assess the effects of social incentives on pro-environmental behavior, the latter are, however, often studied separately (see S10 Table). In addition, the effectiveness of social incentives that promote pro-environmental behavior has not yet been sufficiently investigated in the literature.

While a previous meta-analysis study focused on the crucial role of social influence on resource conservation [13], our study covers other social incentives (network and trust). Our proposed categorization of social incentives is supported by the fact that besides social influence (i.e., internal and external influence), network factors (i.e., network size, network connection and leadership) and trust (i.e., trust in others and trust in institutions) are important concepts that could strengthen social norms and thus shape individual behaviors in a desirable manner, as previously discussed. We contribute to the literature by addressing all these groups of social incentives together to answer the following question: Which social incentives are more effective in encouraging pro-environmental behavior? In response to this question, we conducted a meta-analysis to provide an empirical insight into these seven groups of social incentives. Note that meta-analysis is a well-known statistical technique that helps combine the results of multiple scientific studies, establish an evidence-based practice, and resolve uncertain research outcomes [39]. We took the impact of the aggregation level into account by organizing the seven social incentive groups into three higher aggregated social groups (i.e., social influences, network and trust) and investigated their relative relevance with respect to the metadata. The purpose is to quantify the strength and relevance of social incentives regarding pro-environmental behavior and give some policy recommendations.

The rest of the paper proceeds as follows. In Section 2, we describe the meta-analysis results. Section 3 is devoted to discussions and a conclusion. Section 4 describes data collection, descriptive statistics and the methodology used. In this section, heterogeneity and publication bias problems are also checked to warrant the robustness of the analysis. Since heterogeneity probably exists between studies, the meta-regression model is adapted to take this heterogeneity into account.

## Materials and methods

### Data collection

**Fig 1:**
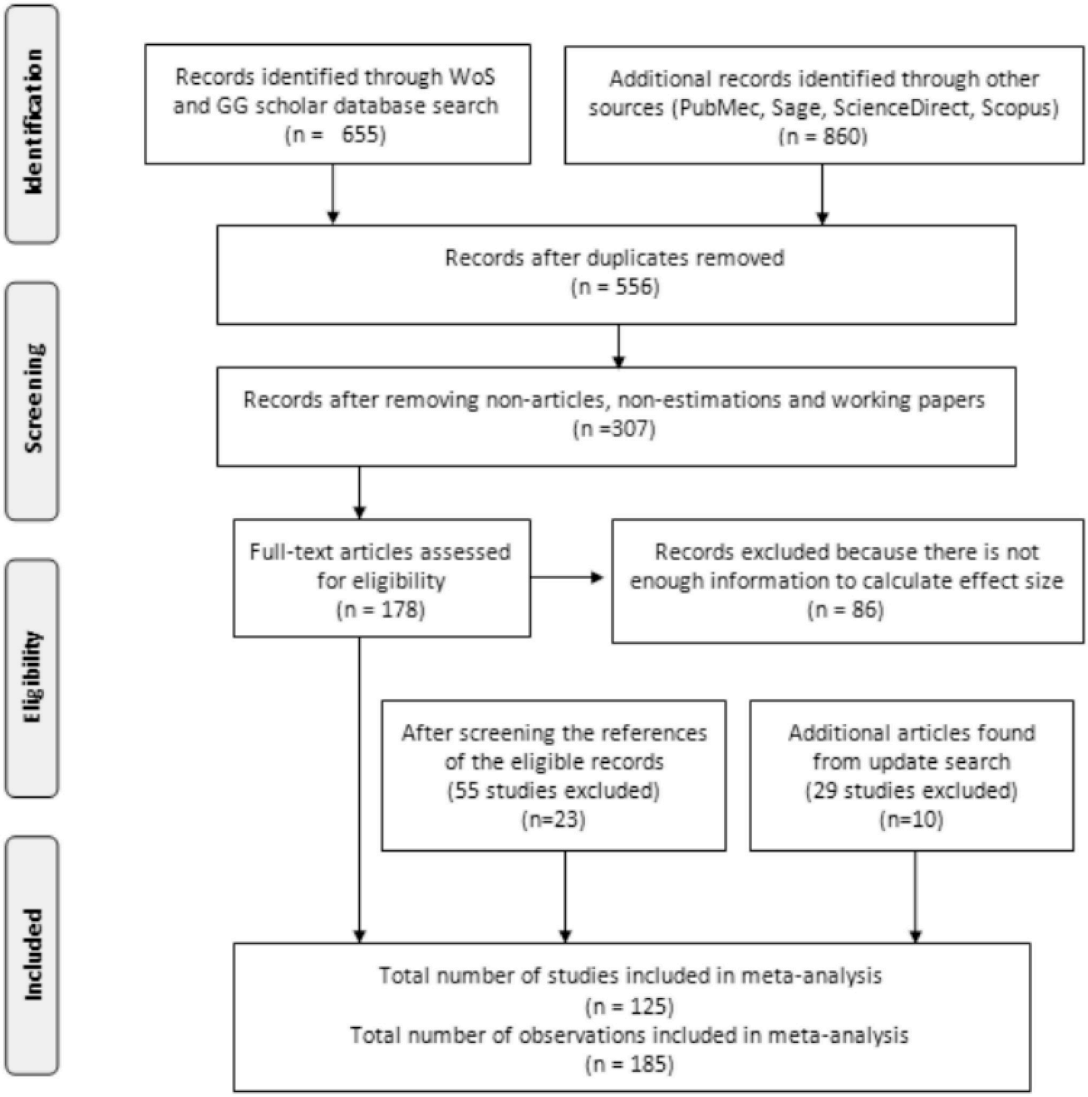
PRISMA flow diagram of data collection.

The dataset in our study was built using the Web of Science, Google Scholar, PubMed, SagePub, and ScienceDirect databases and some other relevant journal websites. A PRISMA flow diagram of data collection is presented in Fig 1. We used keywords to search for related pro-environmental behavior and social incentives: “pro-environmental behaviors”, “sustainable behaviors”, “environmental conservation”, “green behaviors”, “social incentives”, “social intervention”, “social influence”, “social interaction”, “norms”, “nudges”, “networks”, “network structures”, “group size”, “network size”, “network connections”, “network density”, “leader”, “leadership”, “social expectation”, “social comparison”, “peer influence”, “trust”, “social trust”, “institutional trust”, “trust in others” or “trust in government” and all possible combinations of these keywords (All information about these keywords and search strategy for the Web of Science database is presented in Table 1). We also took both the UK and US English into account when performing our keyword searches (e.g., behaviors and behaviours.) and with/without plurals and Boolean operators (OR, AND, *). With these systematic keyword searches, we collected all the published and unpublished works (1,515 papers). We then did the abstract analysis and excluded all duplicated studies, working papers, books, papers without estimation results and papers with only simulation results. We then put all these papers into our meta-analysis dataset (307 papers). We eliminated all the papers that did not provide enough information to calculate standard errors of effect size (i.e., t-values, p-values, confidence intervals or significance levels). After this step, we obtained 92 eligible studies. We continued screening the references of these eligible studies and found an additional 23 eligible records (in 55 relevant studies).

**Table 1:** Keywords and online search strategy.

Group	Keywords
1	“pro-environmental behaviors” OR “pro-environmental behaviours”
2	“sustainable behaviors” OR “sustainable behaviours”
3	“environmental conservation” OR “green behaviors” OR “green behaviours”
A	“social incentives” OR “social intervention” OR “nudges” OR “social comparison”
B	“personal norms” OR “attitudes” OR “instrinsic motivation”
C	“social norms” OR “social expectation” OR “social interaction” OR “peer influence” OR “social influence”
D	“networks” OR “network structures” OR “group size” OR “network size” OR “network connections” OR “network density” OR “leader” OR “leadership”
E	“trust” OR “social trust” OR “institutional trust” OR “trust in others” OR “trust in government”

Data source	Search strategy	Numbers of studies	Numbers of potentially relevant studies	Extend search with Google Scholar and other sources (duplicates removed)	Searches updated (Performed on 25th February 2020)	
					Numbers of additional studies	Numbers of potentially relevant studies
Web of Science	“1” OR “2” OR “3”	2241		280	149	
	“1” AND (“A” OR “B” OR “C” OR “D” OR “E”)	276			54	
	“2” AND (“A” OR “B” OR “C” OR “D” OR “E”)	67			12	
	“3” AND (“A” OR “B” OR “C” OR “D” OR “E”)	206			14	
Total		2790	276 (2790–2514)	556 (276 + 280)	229	89 (229–140)
Total after removing non-estimation, non-article and working papers				307		29
Total after removing articles that do not have enough information to calculate effect size				115		10

Notes: Numbers of potentially relevant studies is total numbers of studies after removing duplicates and not related to pro-environmental behaviors.

An additional update search was conducted in January-February 2020, with an additional ten eligible studies found (among 29 relevant studies). We eventually ended up with 125 studies in the last step. These 125 studies led to 185 observations in our meta-analysis data (in some papers, the authors used more than one social variable to examine the impacts on pro-environmental behaviors). The description of study characteristics is provided in S1 Table. The entire dataset is summarized in S10 Table, and the descriptive statistics in Table 2 and S5 Table.

**Table 2:** A brief summary of the descriptive statistics.

	Definition	Mean	SD
**Dependent variables**			
PCC	Partial correlation coefficient.	0.136	0.151
Coefficient	Effect size coefficient.	1.682	16.392
**Predictor variables**			
SEpcc	Standard error of the partial correlation coefficient.	0.053	0.043
SE	Standard error of the effect size coefficient.	0.827	5.767
*Social influence factors*			
Internal social influence	= 1 if there is the presence of internal influence, such as personal norms, attitudes or intrinsic motivation.	0.297	0.458
External social influence	= 1 if there is the presence of external social influence, such as norms, peer influence, environmental information treatments, or comparative feedback treatments.	0.297	0.458
*Network factors*			
Network size	= 1 if there is the presence of environmental network (group) size or friend (neighbor or work) group size.	0.049	0.216
Network connection	= 1 if there is the presence of network (social, neighborhood, community or environmental group) ties.	0.103	0.305
Leadership	= 1 if there is the presence of a group leader or leadership support in pro-environmental behaviors.	0.065	0.248
*Trust*			
Trust in institutions	= 1 if there is the presence of individual trust in institutions (government, leaders or public/environmental institutions).	0.076	0.266
Trust in others	= 1 if there is the presence of individual trust in others (family, friends, neighbors or community).	0.114	0.319
**Control variables**			
*Differences between geographical regions*			
America	= 1 if study was conducted in the Americas.	0.248	0.433
Asia & Pacific	= 1 if study was conducted in Asia and the Pacific.	0.300	0.459
Europe	= 1 if study was conducted in Europe.	0.300	0.459
MEA	= 1 if study was conducted in the Middle East and Africa.	0.043	0.205
Multiple countries	= 1 if study was conducted in more than one country.	0.103	0.305
*Difference in model specifications*			
Presence of demographic control	= 1 if study was controlled for household size, age or gender.	0.502	0.501
Presence of education control	= 1 if study was controlled for participants’ education level.	0.327	0.470
Presence of income control	= 1 if study was controlled for household income, wages or country GDP.	0.360	0.481
*Types of data collection method*			
Experiment	= 1 if study was conducted using field experiment or laboratory experiment.	0.120	0.326
Direct contact	= 1 if study was conducted using face-to-face interview, telephone interview or questionnaires.	0.453	0.499
Indirect contact	= 1 if study was conducted using online survey or mail (email) survey.	0.311	0.464
Census data	= 1 if study was conducted using census data.	0.114	0.319
*Types of population*			
Employed	= 1 if study’s population is employers or employees.	0.097	0.297
Demographic-related	= 1 if study’s population is students, teachers, children or residents.	0.200	0.401
Household	= 1 if study’s population is households.	0.502	0.501
Agriculture-related	= 1 if study’s population is farmers, fishers or forest users.	0.081	0.273
Others	= 1 if study’s population is car-drivers, internet users, investors, landowners, tourists or countries.	0.118	0.324
Publication year	Study’s publication year.	22.808	5.263

Notes: The detailed definitions of dependent and explanatory variables are provided in Table 1. The detailed descriptive statistics is given in the S5 Table.

### Dependent variable

Pro-environmental behavior is defined as “behavior that consciously seeks to minimize the negative impact of one’s actions on the natural and built world” [40]. Pro-environmental behavior in our meta-analysis is measured across 13 different types of pro-environmental behaviors identified from the literature (including pro-environmental behaviors, pro-environmental intentions, energy consumption, energy conservation, water consumption, water conservation, recycling, environmental conservation, environmental program, environmental groups, green consumption, resource extraction, and workplace pro-environmental behaviors). The definitions of dependent variables are provided in S3 Table. The detailed descriptive statistics of 13 different types of pro-environmental behaviors are reported in S5 Table. We observed that social incentives are more efficient in promoting pro-environmental intention and green consumption but less efficient in encouraging resource conservation.

The effect sizes are the estimated coefficients in the selected studies. The standard errors of the effect sizes are the standard errors of the coefficient estimates. When a paper did not report the standard errors, we calculated them using the corresponding reported t-statistic, the (two-sided) p-value, the confidence interval or the significance level. For papers that only reported insignificant results, we computed the standard errors at a 50% significance level [13, 41]. In order to account for heterogeneity in effect sizes across studies, we performed weighted meta-regression (see details in Meta-regression section). In order to account for heterogeneity in effect sizes across studies, we performed weighted meta-regression (see details in Meta-regression section). In order to summarize and compare the results from various studies, in addition to the effect sizes included in the weighted regressions, we also used the partial correlation coefficients (PCCs) that are often used in meta-analysis in order to make different comparable studies which are based on different units of measurement [42, 43]. The PCC can be calculated by the t-statistic of the reported regression estimate *t*_*ij*_ and the regression degrees of freedom *df*_*ij*_: $PCC_{ij}=\frac{t_{ij}}{\sqrt{t_{ij}^{2}+df_{ij}}}$, where *i* is the observation *i* in the study *j* [44]. The standard errors of the PCC are calculated using the formula: $SEpcc_{ij}=\frac{PCC_{ij}}{t_{ij}}$. We did not explore the possibility of using the standardized effect sizes to compare the magnitudes of variable coefficients because it leads to a reduction in the number of observations due to missing data on standard deviations of dependent variables and regressors.

### Predictor variables

We identified seven different groups of social incentives that can enhance pro-environmental behavior: social influence (including internal and external influence); network factors (including network size, network connection and leadership); and trust (including trust in others and institutions). The detailed definitions of these seven social dummies are given in S4 Table. The diagram of these seven groups of social incentives is presented in S1 Fig.

In our study, we consider *external social influence* as external motives (e.g., extrinsic motivation, the expectation of others or social norms) that help motivate people to behave toward the environment. In contrast, *internal social influence* is defined as internal motives (e.g., attitudes, personal norms or intrinsic motivation) that could encourage people to take actions to protect the environment. It should be noted that “social comparative feedback” is an external factor that could internally motivate individuals by generating self-evaluation (i.e., individuals evaluate themselves). Self-enhancement could also encourage people to act and sustain their behaviors over time [45–47]. In other words, competitive behaviors drive self-evaluation, and the necessity of such an evaluation is based on the comparison with other people [45]. On the other hand, since people observe social behaviors (i.e., social norms) through comparative feedback, they could change their behaviors to fit in with a group (i.e., conformity or internalization) or take the social beliefs as their personal beliefs (i.e., group or belief polarization). Nevertheless, “social comparative feedback” is an external factor as it provides information treatment (i.e., feedback information) to individuals in the treatment group, leading us to consider “social comparative feedback” as an external social influence in our meta-analysis. We have the following hypotheses:

**H1a:** The presence of internal social influence could positively impact pro-environmental behaviors.
**H1b:** The presence of external social influence could positively impact pro-environmental behaviors.

Concerning network, each individual is represented by a social unit (or node). Social units are linked together through social relationships such as friendship or acquaintanceship [48]. *Network connection* is the degree of connection or the relationship between individuals and others, including friends, neighbors, environmentalists and environmental organizations. A strong network connection comes from the solid ties or interactions among individuals inside the network (i.e., a dense network), which is equivalent to what is referred to as the “good sense of community”. The latter means that individuals frequently interact with each other and that they care more about their community. Several empirical studies have shown that the “good sense of community” can directly shape individuals’ behaviors and force them to care more about environmental issues [49–51]. *Network size* captures the number of friends, neighbors or co-workers involved in pro-environmental actions or environmental associations that individuals participate in. *Leadership* captures the presence of environmental leaders that can influence individuals’ pro-environmental behaviors. We have the following hypotheses:

**H2a:** A stronger network connection (i.e., higher frequency of interactions) has a positive impact on pro-environmental behaviors.
**H2b:** A larger network size (i.e., more individuals in a network) has a positive impact on pro-environmental behaviors.
**H2c:** The presence of leadership could help promote pro-environmental behaviors.

Finally, *trust in institutions* is defined as individuals’ trust in government, institutions, or leaders. *Trust in others* is defined as a social trust or individuals’ trust in friends, neighbors and family. We have the following hypotheses:

**H3a:** A higher trust in institutions could positively impact pro-environmental behaviors.
**H3b:** A higher trust in others could positively impact pro-environmental behaviors.

The correlation matrix of these seven social incentives, provided in S2 Table, indicates that the multicollinearity problem is not present in the data. The descriptive statistics are given in Table 2 and S5 Table for more detailed descriptive statistics. The descriptive statistics suggest that internal social influence appears to be more effective than other social incentives in encouraging pro-environmental behavior. Table 2 also shows that the two social incentives commonly studied in the existing literature are internal and external social influence, which accounted for about 60% of the observations. Meanwhile, the less commonly used social incentive factor is network size, with 4.92% of the observations.

### Control variables

In order to address the issue of geographical difference or other factors correlated with geographical regions (i.e., regional heterogeneity), we first controlled for the difference between regions (including America, Asia & Pacific, Europe, and Middle East & Africa) and also studies that were conducted on multiple countries. The list of countries is provided in S1 Table. Secondly, we accounted for the heterogeneity across different specifications regarding control variables (demographic characteristics, education, income, etc.). Thirdly, we included data collection methods used in the studies, such as experiment, direct contact, indirect contact and census data. Fourthly, we also controlled the types of targeted populations to capture the differences among households, demographic-related populations (students, teachers, children or residents), agriculture-related, employed, and other population groups. Finally, we included the study’s publication year to capture the time trend of pro-environmental behavior estimates since we observed an increase in effect size (PCC) of the reported pro-environmental behavior across publication year (see S3 Fig). Descriptive statistics of the control variables are reported in Table 2 and S5 Table, respectively.

According to the results of the study’s characteristics reported in S1 Table, we observed that most of the selected studies were done in America, with 37 studies in all. However, a smaller number of studies were conducted in the Middle East and African countries. On average, only 62 papers in our study controlled for demographic variables (including household size, age or gender), and only 40 papers controlled for education and income variables (including participants’ education levels and income or wages). Most of our studies were conducted using direct contact (including face-to-face interviews, telephone interviews and questionnaires). The most common population used to investigate pro-environmental behavior was households, with 65 studies in all. A smaller number of studies targeted agriculture-related populations such as farmers, fishers or forest users.

### Publication bias

**Fig 2:**
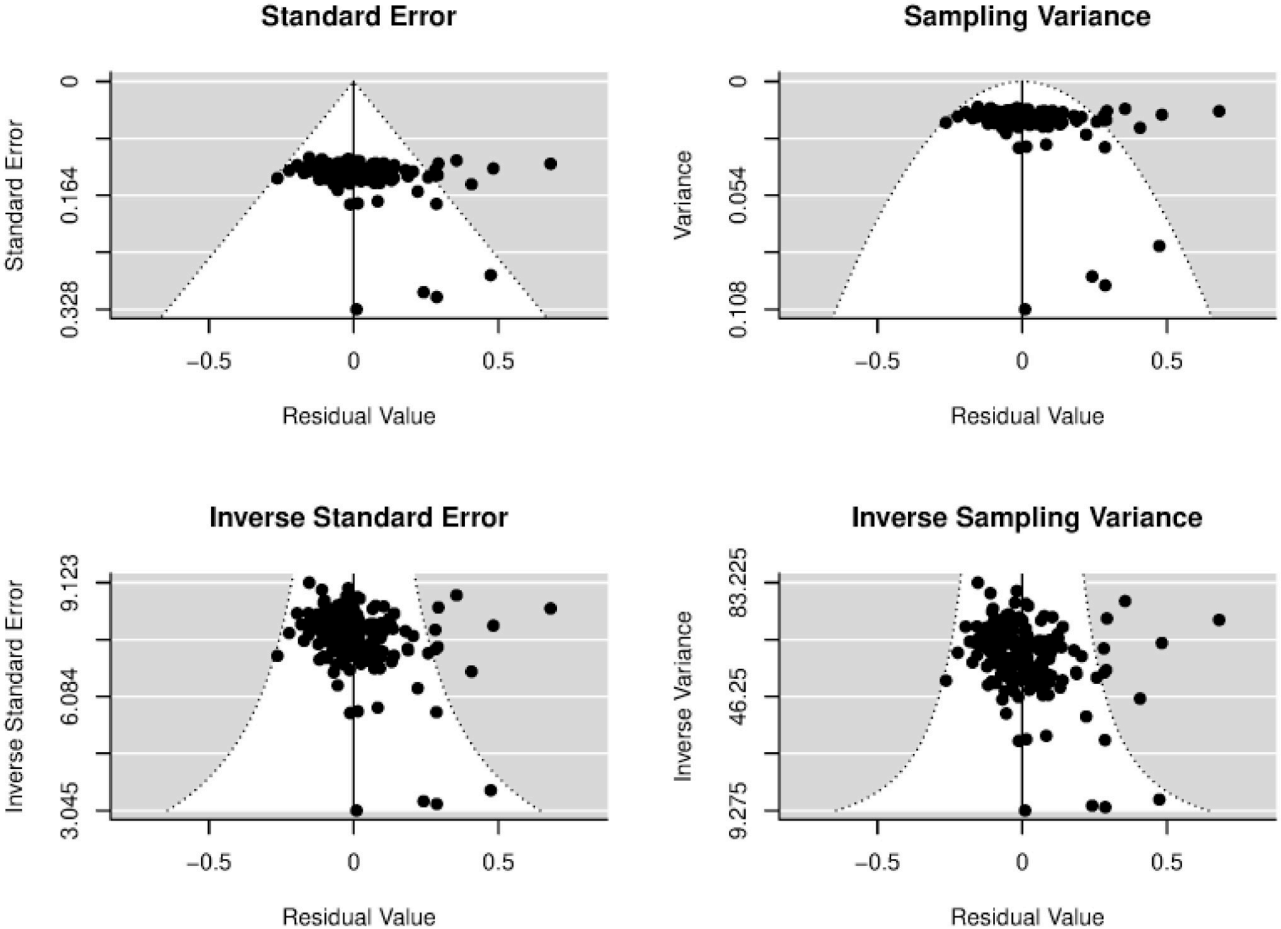
Funnel plot for publication bias.

Fig 2 shows the funnel plot with the regression residuals compared to their corresponding standard errors. This graph is used to access the publication bias [52, 53]. The latter corresponds to a type of bias that refers to the distortion of empirical data representation on a subject [54]. For instance, empirical data is distorted because reviewers of scientific journals tend to accept studies with significant positive effects rather than negative or insignificant ones. In the absence of a publication bias, we would expect that the majority of the observations would fall inside of the pseudo-confidence region with bounds $\hat{\alpha }\pm 1.96SE$, where $\hat{\alpha }$ is the estimated effect of the mixed-effects model and *SE* is the corresponding standard error value [53]. Egger’s regression test for funnel plot asymmetry: z-stats = 3.256, p = 0.001 suggests that asymmetry presents in the funnel plot [55], implying that positive estimates may be preferably selected for publication. We should therefore focus on the formal methods of detection of and correction for publication bias. According to the literature, we should regress the estimated effect size on its standard error [56]:

$$\begin{array}{c} PCC_{ij}=\beta_{0}+\beta_{1}SEpcc_{ij}+\epsilon_{ij}, \end{array}$$ (1)

where the coefficient *β*_0_ denotes the overall (average) effect size and *β*_1_ measures the magnitude of the publication bias.

Eq (1) is probably heteroskedastic because of, for example, different measurements between studies and dependence of estimates within a study due to multiple estimates per study. Thus, we apply the weighted least squares to the following multivariate mixed-effect model with the weights defined by the standard errors of the effect size (1/*SEpcc*_*ij*_) [57, 58]:

$$\begin{array}{c} \frac{PCC_{ij}}{SEpcc_{ij}}=\beta_{1}+\beta_{0}\frac{1}{SEpcc_{ij}}+\alpha_{j}\frac{1}{SEpcc_{ij}}+\frac{\epsilon_{ij}}{SEpcc_{ij}}, \end{array}$$ (2)

where *α*_*j*_ is the study-level random effect and $\mu_{ij}\equiv \frac{{∊}_{ij}}{SEpcc_{ij}}$ is the estimate-level disturbances.

Estimation results of Eq (2) are provided in S6 Table. The results suggest that the null hypothesis *β*_1_ = 0 is rejected at the 10% significance level, meaning that there is some evidence of funnel asymmetry. The positive constant suggests that publication selection is favorable to positive effects. This result is also in line with the results of Egger’s regression test.

### Forest plots and heterogeneity

**Fig 3:**
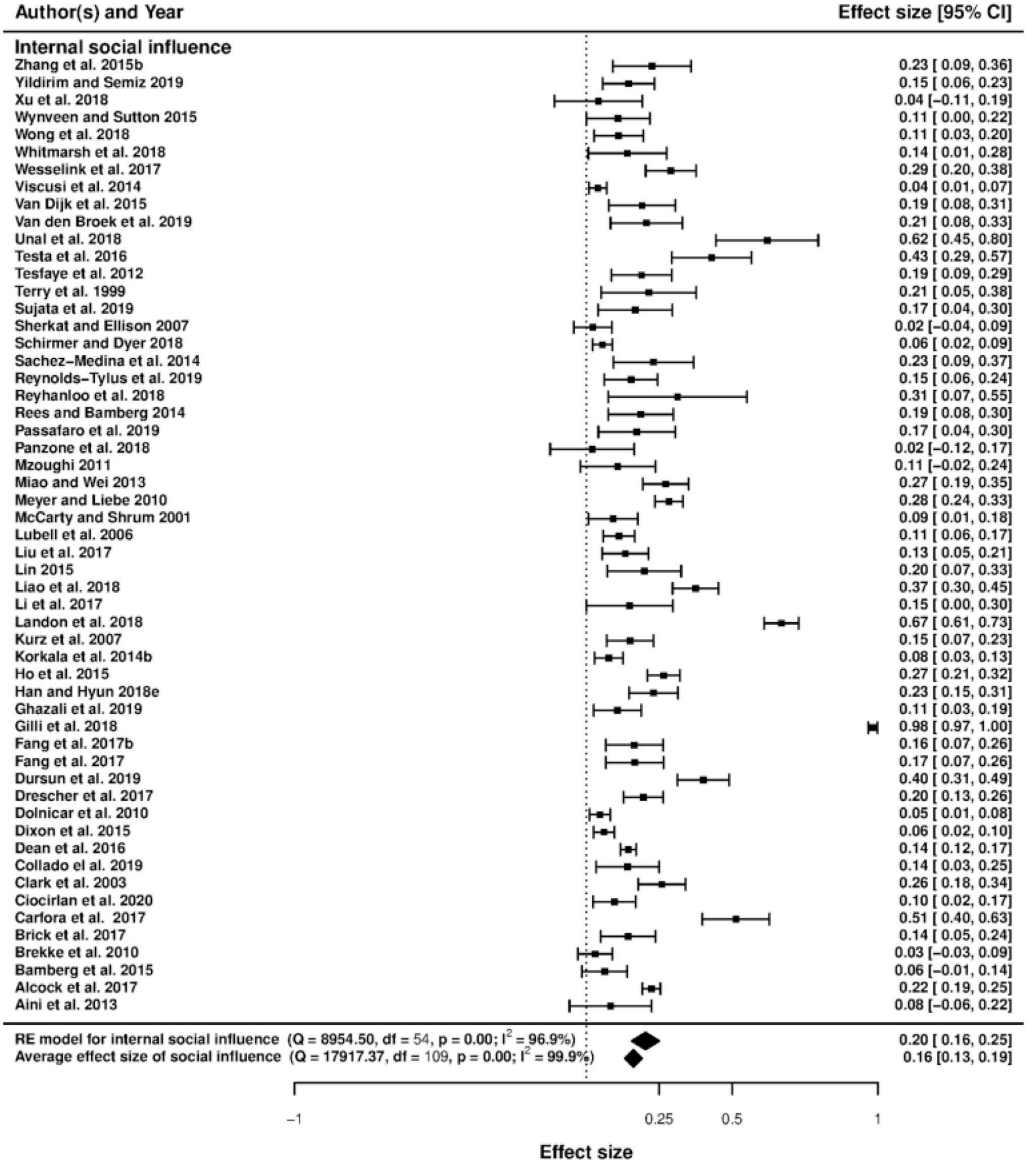
Forest plot of internal social influence.

**Fig 4:**
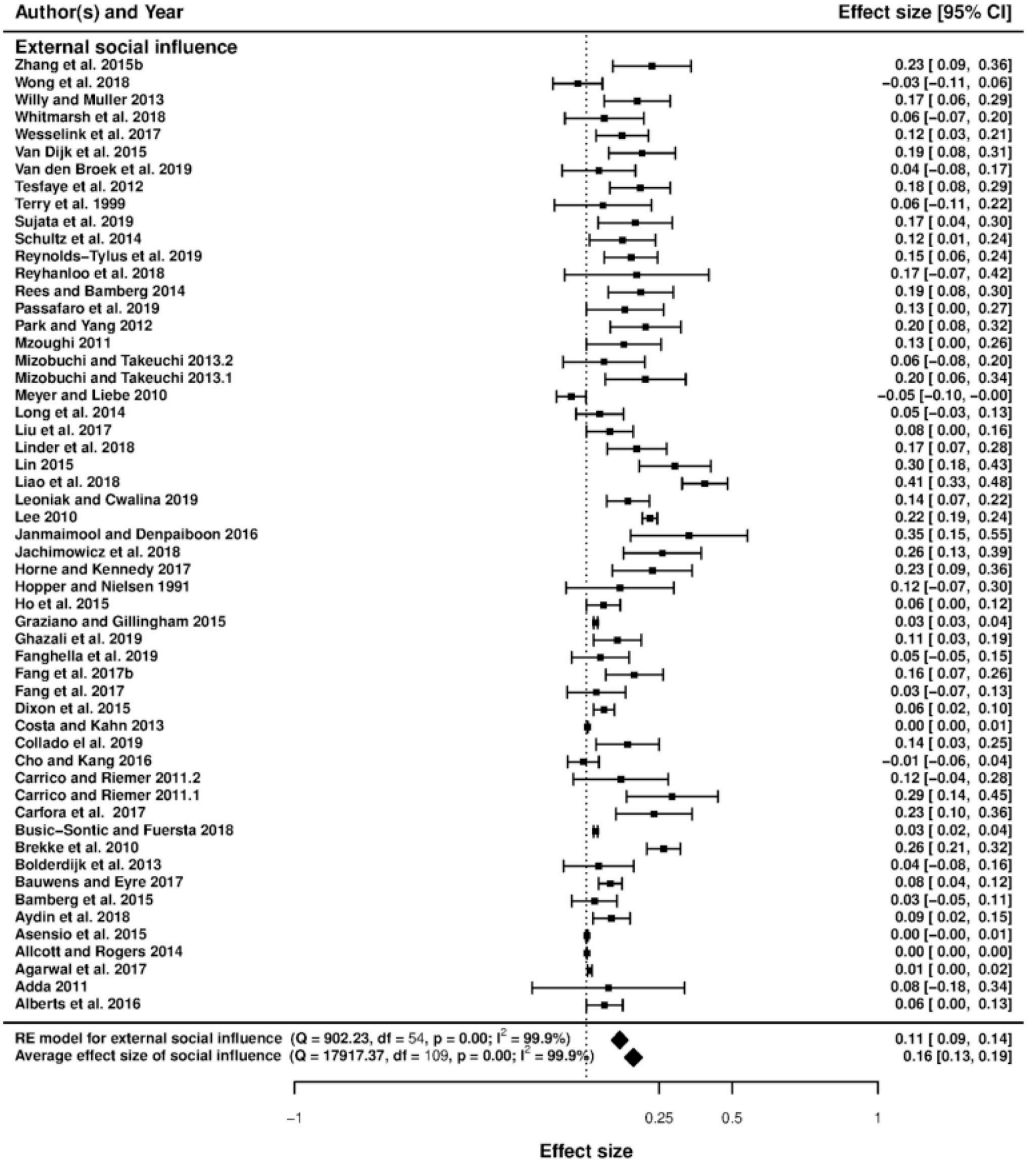
Forest plot of external social influence.

**Fig 5:**
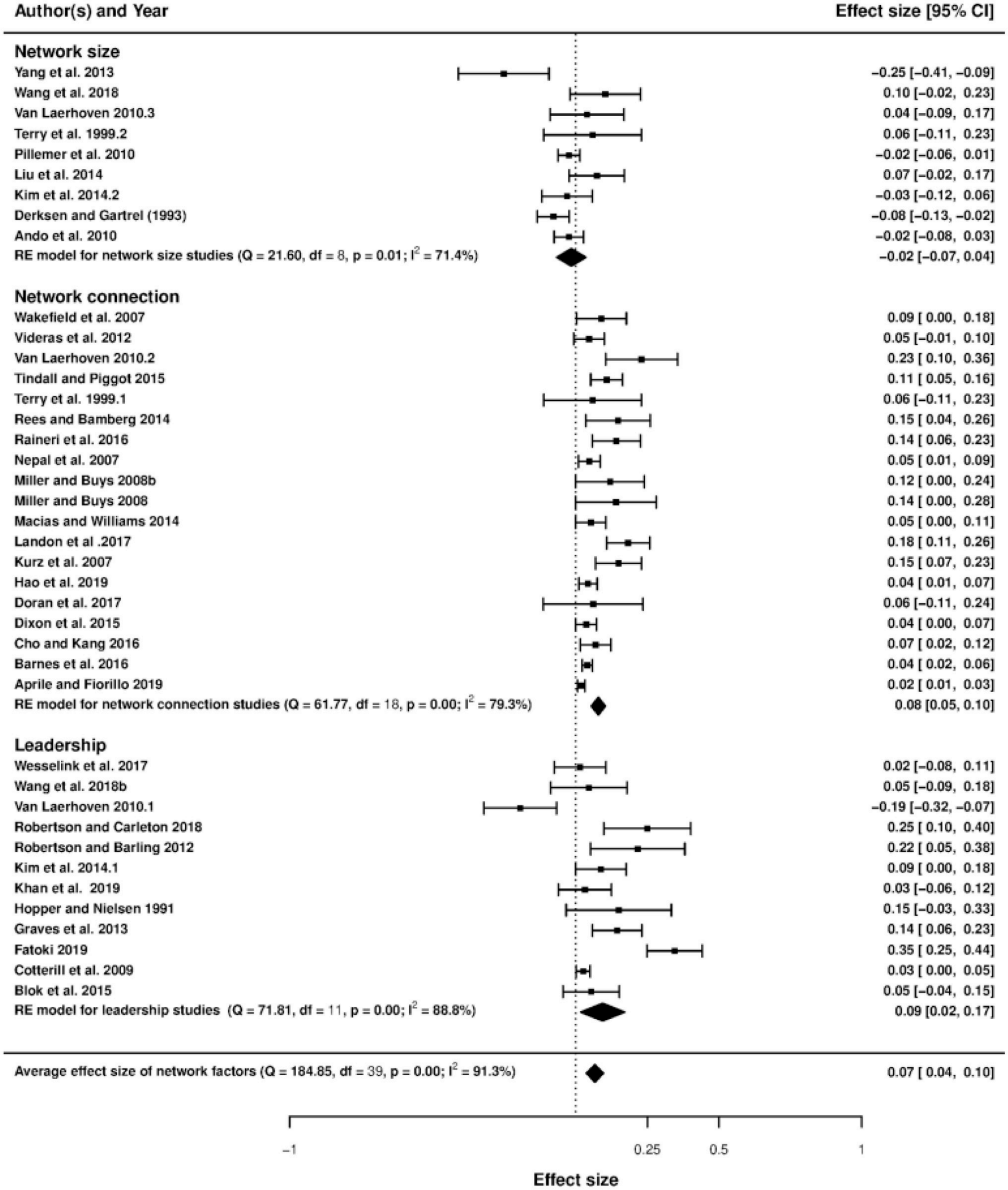
Forest plot of network factors.

**Fig 6:**
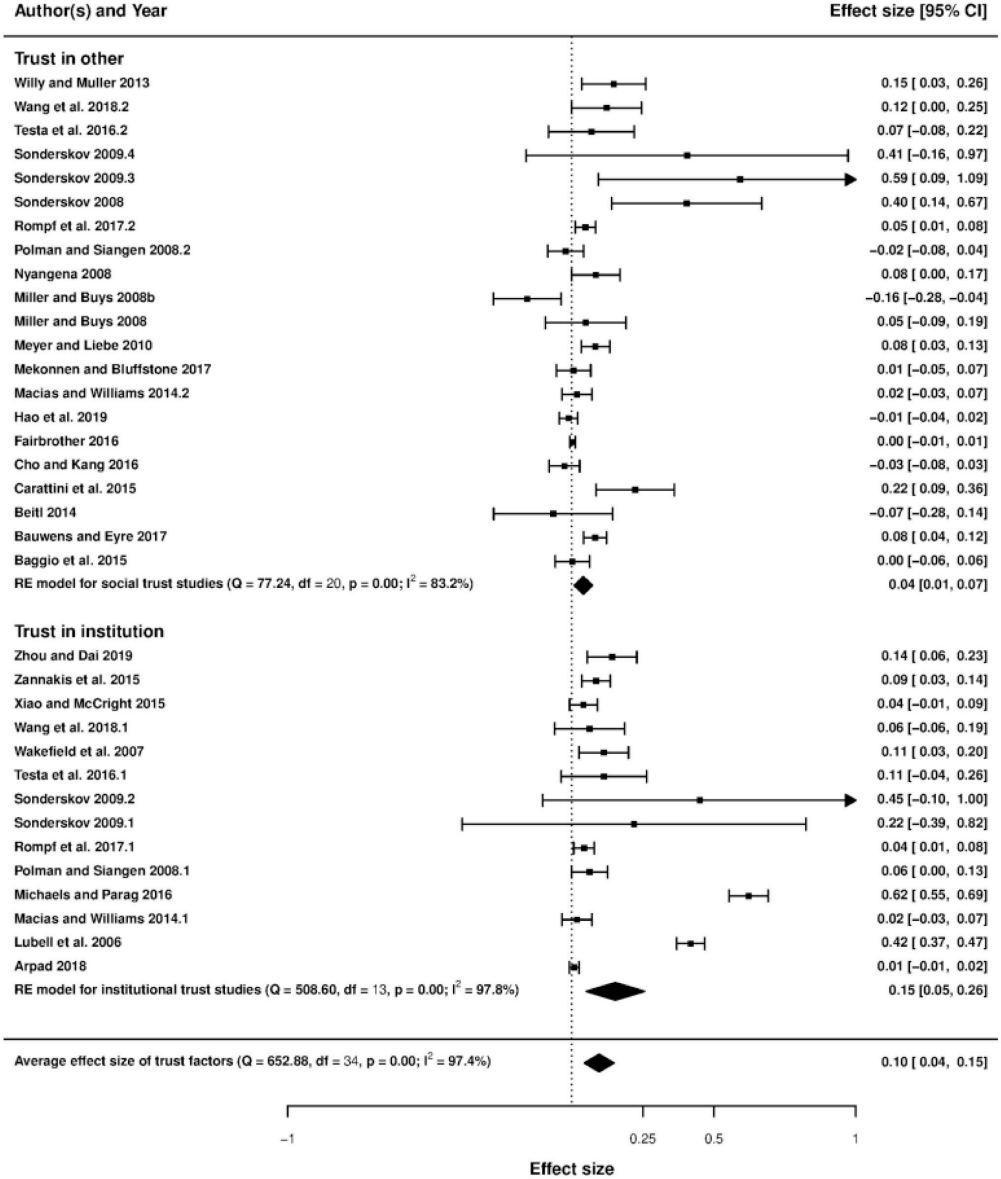
Forest plot of trust factors.

Figs 3–6 display the forest plots of effect sizes and their precision. The forest plot illustrates the results of several studies with horizontal lines showing the confidence interval for each study and a mark to show the point estimate. It provides a visual presentation of the amount of variation between the results of the studies, as well as an estimate of the overall result of all the studies together [59]. Studies are divided into seven sub-groups of social incentives, i.e., internal social factor (Fig 3), external social factor (Fig 4), network factors including network size, network connection and leadership (Fig 5) and trust, including trust in others and trust in institutions (Fig 6). The overall effect size of each sub-group (indicated by a diamond) is also at the bottom of each study subset.

The overall effect size of all studies is first calculated by fitting a random-effect model (*β* = 0.132, 95%*CI* = [0.107, 0.157]). When the between-study variance is non-zero, the random-effect model for meta-analysis is a well-known approach to account for heterogeneity among studies. The random effect model is fitted using the restricted maximum likelihood, which is the most recommendable property [60]. The random effect model is as follows: *PCC*_*ij*_ = *β* + *α*_*j*_ + *ϵ*_*ij*_, where *α*_*j*_ is the study-specific random effect; *ϵ*_*ij*_ is the error term; *β* is the overall effect size. The regression is weighted by a weight equal to 1/(*τ*^2^ + *v*_*i*_), where *v*_*i*_ is individual variance and *τ*^2^ is between-study variance, typically preferred to as the amount of heterogeneity [61]. This suggests that social incentives are generally good at encouraging pro-environmental behavior. The Cochran *Q*-statistic for heterogeneity, which is the weighted deviations related to the summary effect size, is also calculated [62]. The *Q*-test statistic *Q*(*df* = 184) = 18867.97 with *p* < 0.001 suggests that heterogeneity exists in our meta-analysis (statistically significant between-study variance).

In addition to the heterogeneity of study effect sizes, we applied the moderator analysis with several control variables: differences between regions, differences in specification (presence of demographic, education and income variables), data collection methods (field experiment, direct and indirect method, and census data), types of population (households, employed, agriculture-related, etc.) and publication year (*Q*-test for moderator: *QM*(*df* = 16) = 40.66, *p* < 0.001). We also fit the mixed effect model with restricted maximum likelihood and with the Cochran *Q*-stat = 7568.11, df = 168, *p* < 0.001 (the mixed-effect model is $PCC_{ij}=\beta_{0}+\beta_{1}SEpcc+\sum_{l=1}^{L}\delta_{l}Z_{ijl}+\alpha_{j}+\varepsilon_{ij}$, where *δ*_*l*_ is the fixed slope, *α*_*j*_ is the study-specific random effect, *ε*_*ij*_ is the error term and *β*_1_ is the publication bias).

### Meta-regression model

We adopted the meta-regression analysis method to further shed light on the ‘black box’ of our meta-analysis results [63, 64]. We used the following meta-regression model (where *i* = 1, 2, …, *N* and *j* = 1, 2, …, *M* stand for observations and studies, respectively):

$$\begin{array}{c} y_{ij}=\beta_{0}+\beta_{1}x_{ij}+\sum_{k=1}^{K}\gamma_{k}SD_{ijk}+\sum_{l}^{L}\delta_{l}Z_{ijl}+\alpha_{j}+\varepsilon_{ij}, \end{array}$$ (3)

where *y*_*ij*_ is either the effect size coefficient (*Coef*_*ij*_) or the partial correlation coefficient (*PCC*_*ij*_) of observation *i* and study *j*. Note that *x*_*ij*_, included here to account for the publication bias, corresponds to the standard error of *Coef*_*ij*_ (*SE*) or the standard error of *PCC*_*ij*_ (*SEpcc*) depending on the considered regression. A positive (negative) value of *β*_1_ implies a positive (negative) publication bias. *SD*_*ijk*_ are the social incentives dummies including internal social influence, external social influence, network size, network connection, leadership, trust in others and trust in institutions (there are *K*=7 social incentives dummies, network size being the base category). *Z*_*ijl*_ is a vector of study-level characteristics (*L* = 18 control variables). In Eq (3), the meta-regression coefficients *δ*_*l*_ represent the bias related to *L* variables including differences between geographical regions, model specifications (demographic, education and income factors), types of study (field experiment or laboratory experiment, etc.), types of population and publication year. A positive (negative) value of *δ*_*l*_ implies a positive (negative) bias. Finally, *ε*_*ij*_ is the meta-regression model error. Note that because of the presence of predictor variable dummies and control variable dummies, the intercept of the meta-regression above (*β*_0_) cannot help to separately identify the overall effect size and the values of the base categories of these groups of dummies.

Because most of the primary literature uses different data sets, different dependent variables, different types of data collection methods and different sample sizes, it is reasonable to suspect that the meta-regression error is likely to be heteroskedastic (see S2 Fig for the plot of partial correlation coefficient vs. squared root of study’s sample size). We therefore estimated the model using weighted least squares (WLS) with weights given by 1/*e*_*i*_ (*e*_*i*_ is the observation *i*’s standard errors). When the individual standard error is unknown, the model is estimated using weighted least squares (WLS) with weights given by $1/\sqrt{N_{i}}$ where *N*_*i*_ is the study’s sample size. When the individual standard error *e*_*i*_ is known, the heteroskedasticity can also be corrected by weighted least square regression with weights given by 1/*e*_*i*_ [64, 65].

In summary, we performed the following two regressions with two different dependent variables (*PCC* or *Coef*): (1) WLS with weights given by 1/*e*_*i*_; and (2) the mixed-effect model with weights given by 1/(*τ*^2^ + *v*_*i*_), where *v*_*i*_ is individual variance and *τ*^2^ is between-study variance, typically preferred as to the amount of heterogeneity [61]. Standard errors are calculated using bootstrap with 2000 replications. The estimation results are provided in Table 2 and result with all control variables in S7 Table.

Finally, to investigate the impact of the aggregation level of social incentive factors, we organized the seven social incentive groups into three higher aggregated social groups (i.e., social influences, network and trust). We fit the same model in Eq (3) using these three social incentives dummies (the network group being the base category). To compare our model of seven social incentives dummies (column 4, Table 3) with that of three social incentives dummies (column 6), we applied the Wald test with the null hypothesis of the equality between the coefficients of internal and external social influence dummies, equality between coefficients of network connection, leadership and network size (i.e., the regression intercept), and equality between trust in institutions and trust in others dummies. The Wald test statistic *χ*^2^(4) = 17.35 with *p* = 0.0016 suggests that the model with seven social incentives dummies is preferable. The computed statistic of an alternative test (likelihood ratio test) is *χ*^2^(4) = 16.62 with *p* = 0.002, also suggesting that the unrestricted model (i.e., model with seven social incentives dummies) is preferable. Consequently, our proposed model with seven social incentives dummies is better than those with three higher-aggregated social incentives dummies. Moreover, in order to compare the magnitude of the impacts of the seven social incentives dummies for our model, we calculated the corresponding standardized coefficients:

$$\begin{array}{c} {\tilde{\gamma }}_{k}={\hat{\gamma }}_{k}\frac{s(SD_{ijk})}{s(PCC_{ij})}, \end{array}$$ (4)

where ${\hat{\gamma }}_{k}$ is the estimated coefficient of predictor *k*, *s*(*SD*_*ijk*_) and *s*(*PCC*_*ij*_) are the sample standard deviation of the predictor *k* and the dependent variable (*PCC*), respectively. Standardized coefficients for other control variables are similarly defined. The standardized coefficients are reported in Table 3 (column 5).

**Table 3:** A brief summary of the meta regression results.

Variables	Coef		PCC			
	Weighted least squares	Mixed-effect model	Weighted least squares	Mixed-effect model, seven social incentives	Standardized coefficient of Model (4)	Mixed-effect model, three social incentives
	(1)	(2)	(3)	(4)	(5)	(6)
**Social influence**						0.058***
						(0.017)
Internal social influence	0.230	0.262***	0.167***	0.154***	0.8454	
	(0.144)	(0.068)	(0.062)	(0.043)		
External social influence	0.104	0.158**	0.062	0.085*	0.4680	
	(0.143)	(0.069)	(0.062)	(0.045)		
**Network factors**						
Leadership	0.046	0.125	0.061	0.053	0.1479	
	(0.196)	(0.087)	(0.078)	(0.057)		
Network connection	0.089	0.174**	0.025	0.091**	0.3444	
	(0.167)	(0.077)	(0.065)	(0.045)		
**Trust**						-0.004
						(0.029)
Trust in institutions	0.208	0.229***	0.091	0.110*	0.3445	
	(0.191)	(0.082)	(0.069)	(0.062)		
Trust in others	0.045	0.074	-0.029	0.030	0.1133	
	(0.157)	(0.079)	(0.066)	(0.047)		
**Control variables**						
*Difference between regions* (Europe as baseline)						
MEA	0.174	0.138*	0.148**	0.119**	0.2901	0.103*
	(0.176)	(0.078)	(0.070)	(0.062)	(0.066)	
Presence of demographic variables	-0.203***	-0.072*	-0.110***	-0.076**	-0.4571	-0.074**
	(0.073)	(0.064)	(0.028)	(0.034)		(0.035)
SE (or SEpcc)	1.951***	1.234***	0.899**	1.123***		1.101***
	(0.134)	(0.179)	(0.455)	(0.381)		(0.373)
Intercept	-10.010	-11.941*	-3.458	-5.542		-6.606
	(9.938)	(6.248)	(5.445)	(3.597)		(5.330)
	-8.383	-12.099*	-3.245	-5.861		-6.606
	(12.144)	(6.263)	(5.689)	(3.593)		(5.330)
Observations	185	185	185	185		185
Studies	125	125	125	125		125

Notes: Meta-regressions with effect size coefficient or partial correlation coefficient as dependent variables. All the columns are obtained from regressions using seven social incentive groups (network size as the base category), except the last one that is based on the regression using three higher-aggregated social incentive groups (network as the base category). Full estimation results with all control variables are given in the S7 Table. Weighted least squares are estimated with weights equal to 1/SE (or 1/SEpcc). In the multivariate mixed-effect model, the weight is calculated using 1/(*τ*^2^+ *v*_*i*_), where *v*_*i*_ is individual variance and *τ*^2^ is between study-variance or typically called the amount of heterogeneity. The Wald test of Model in column 4 vs. Model in column 6 is *χ*^2^(4) = 17.35 with *p* = 0.0016, suggesting that Model in column 4 is preferable. Bootstrap standard errors with 2000 replications are in parentheses.

*p<0.1;

**p<0.05;

***p<0.01.

## Results

Based on the existing literature, as mentioned above, we divided our discussions about the emergence of pro-environmental actions into seven groups of social incentives: internal and external social influence, network factors (network size, network connection, and leadership) and trust (trust in others and trust in institutions) [21, 66]. This result leads to seven social incentive dummies used in the meta-regression (network size being the base category). The forest plots in Figs 3–6 show that internal and external social influence, trust in institutions and network connection are key significant factors that could be used to encourage pro-environmental behavior. Our summarized results (Table 3, column 4, where the mixed-effect model is applied using partial correlation coefficients) suggest that the effect of internal and external social influence, network connection and trust in institutions on pro-environmental behavior are positive and statistically significant, meaning that Hypotheses H1a, H1b, H2a and H3a are validated. On the contrary, leadership and trust in others do not significantly affect pro-environmental behavior, meaning that Hypotheses H2c and H3b are not validated. The standardized coefficients (column 5) suggest that internal social influence is the most effective social incentive (since its value is the highest), followed by external social influence, network connection and trust in institutions.

### Social influence

Our meta-regression results in Table 3 show that there is a positive and significant impact of external social influence on pro-environmental behaviors at a 10% significance level. The forest plot in Fig 4 suggests that the overall effect of external social influence is positive, even if some studies have reported negative results. This result means that the presence of external social influence is overall in favor of encouraging pro-environmental behaviors. For instance, several studies suggested that individuals who gain insights about environmental issues and receive recognition from their peers through “social comparative feedback” (e.g., household energy consumption report) could change and develop more environmentally-oriented behaviors because of the self-evaluation/-enhancement process [45, 67]. Therefore, external social influence is a factor that could positively impact pro-environmental behaviors.

However, in some cases, external social influence could discourage environmental conservation. For instance, one study revealed that comparison feedback might perform poorly in encouraging environmental conservation (e.g., soil conservation) because terracing is a demanding soil conservation practice and farmers have a low perception of environmental issues [68]. Thus, if there is a relatively low individual perception of an environmental issue, external social influence may also fail to promote pro-environmental behaviors. In another example, when asking people how much they are willing to contribute to environmental conservation, individuals will report lower conservation efforts if they know that their profiles and results are invisible to others. People may act collectively but regardless of the demand of the social situation if they know that others cannot observe their actions [69, 70]. For example, if a community could not observe forest owners’ behaviors and if there were no regulator to monitor them, then forest owners would collectively choose deforestation, even with strong social expectations (social norms) that forest conservation is essential for society [71, 72].

Regarding the forest plot in Fig 3, most of the studies in the literature suggest a positive effect of internal social influence on pro-environmental behaviors. In addition to the forest plot, our meta-regression results in Table 3 suggest that internal social influence has a positive and significant effect on pro-environmental behaviors. The standardized coefficients reported in Table 3 (column 5) indicate that the internal social influences that motivate people to change their perceptions and attitudes are significant to promote pro-environmental behaviors (the standardized coefficient is 0.8454 while that of external influence is 0.4680). Thus, internal social motives that help guide people to change their perceptions and attitudes toward a sustainable behavior could be more efficient than the external social influence [16, 17, 20]. For instance, one study has suggested that altering individuals’ perceptions by providing different visual attention to climate information (e.g., global temperature change) could reinforce their beliefs and motivations to take actions to mitigate climate change [73, 74].

### Network factors

Our results suggest that the effect of network connection is relatively strong, with the standardized coefficient equals 0.344 (see the results in Table 3). In one study, the authors showed that network has an indirect impact on ecological health because it helps to share information and knowledge across individuals and to promote cooperation among members of the network [75]. On the other hand, individuals are less likely to take a conservation action because they fear that their neighbors may free-ride on their efforts, such as restoring soil functions or investing in fertility improvements [76, 77]. Thus, a stronger network connection (i.e., a denser network), the main characteristic of network structure, is an effective social incentive to enhance environmental behavioral changes.

Several studies have indicated that larger network size is responsible for weaker network connection or less social interaction because individuals in a society or group only make contact or frequently interact with others living close to them [78–80]. Our forest plot (Fig 5) suggests that network size does not affect pro-environmental behaviors. Meta-regression results also support this finding by indicating that other groups of social incentives positively affect pro-environmental behaviors compared to network size (as the base category). It should be noted that the effect of network size corresponds to the regression intercept. The latter also corresponds to the overall effect size in the meta-regression. Moreover, it represents the effect of the base category for other groups of dummies (more precisely, census data among types of data collection methods and Europe among geographical regions). Consequently, we cannot separately identify the overall effect size, the effect of network size, and the effect of the base category for other dummy groups. A simple meta-regression without variables of interest (i.e., social incentives) and any control variable gives a very rough estimation of the overall effect size (see S6 Table, also corresponding to a test for publication bias). When this meta-regression model is augmented by social incentive dummies (see Table 3, columns 1’ and 3’ using effect size coefficient and partial correlation coefficient as dependent variables, respectively), the intercept may become (significantly or not) negative. This is because the intercept also includes the effect of the base category of social incentives (i.e., network size). Hence, increasing network size will not result in a better environmental outcome, ceteris paribus. By comparing this result with the network connection, we would expect that if an increase in the network size is accompanied by an increased degree of connection between individuals, the adverse effect of network size can be more than offset by the positive effect of connections between individuals, leading to a pro-environmental action. In other words, when requiring this combination of network size and network connection, the point of vigilance must be to observe the necessary condition of an increase in the connection.

In order to have a sustainable network, the presence of a good leader appears to be necessary. This leader is responsible for providing information and keeping people connected. For example, some studies showed that a “block leadership” approach treatment has a positive impact on the recycling rate of households because a leader plays a vital role in sustaining a connection and providing needed information to households within the leader’s network [81, 82]. Block leaders are defined as volunteers who help inform people in their groups about a specific issue. However, the coefficient of leadership in our meta-analysis regression is statistically insignificant compared to the network size (see Table 3). This result is not surprising because, among the positively significant results of leadership, some studies reported the positively small and even negative impact of leadership on pro-environmental behaviors (see the forest plot in Fig 5). For example, one study indicated that the presence of a leader in groups that have the autonomy to craft governance rules for their environmental resource could encourage the group’s collective actions toward resource conservation but discourage resource conservation when groups are subject to rules imposed by others [26]. A detailed analysis of the autonomy to craft governance rules would be interesting but is beyond the scope of this paper. Thus, it would be interesting for future studies to take it into account when studying the impact of leadership on pro-environmental behaviors. We also re-estimated the model by excluding one paper that reported a negative impact of leadership and obtained the same results. We can be confident that an outlier is not present in our data.

### Trust

Our meta-analysis results (Table 3) show that trust in institutions is a driver of pro-environmental behaviors. A lack of trust in government can lead to a negative individual perception of an institutional design/government program and prevent the individual from participating in it. For example, a well-designed agri-environmental contract cannot completely replace a farmer’s trust in government institutions [83]. The existing literature has also suggested that the rate of participation in an environmental program can be increased by motivating people and by maintaining and developing institutional trustworthiness [84, 85].

Regarding the forest plot in Fig 6, trust in others has an overall positive effect on pro-environmental behaviors. Similar to trust in institutions, maintaining trustworthiness between individuals has a positive impact on behavioral changes, as shown by numerous studies [86]. However, our meta-regression results cannot confirm the significant impact of trust in others on pro-environmental behaviors (see Table 3). Some of the existing literature suggests that trust in others may fail to motivate new attitudes about environmental issues and pollution [87]. In their study, the authors argued that a higher level of trust within a close network could cultivate a sense of comfort and security and thus makes people less likely to respond to less immediate and indirectly observable environmental issues. One study indicated that trust in others performing resource conservation behaviors might have a low impact on resource extraction because of the subtractability property of the common resource (i.e., consuming an additional common resource would decrease the available resources for others) [88]. As a result, because of the resource constraint, individuals who trust in others performing resource conservation feel that they have no choice but to harvest whatever the environment provides.

## Robustness checks

Regarding the robustness of our estimation results, we classified pro-environmental behaviors into three different groups, including environmental conservation, environmental consumption and general pro-environmental behaviors. The detailed classification of the dependent variable is reported in S8 Table. Indeed, conservation efforts are more likely to have positive spillovers on others (i.e., positive externalities) and also more likely to be observed by others (i.e., visibility or observability) than consumption efforts (e.g., eating green). We observe that the social incentive factors play a crucial role in promoting environmental conservation rather than environmental consumption and general pro-environmental behaviors (the estimation results are reported in S9 Table).

Alternatively, we checked the robustness by classifying the observed pro-environmental behaviors into two different groups, including high dependency (i.e., pro-environmental behaviors that highly depend on the critical contribution of others to ensure their successes) and low dependency, which is otherwise the pro-environmental behaviors that less likely depend on the critical contribution of others to ensure their success. The detailed definitions of these two classifications are reported in S8 Table. Indeed, similar to public good contributions, some kinds of pro-environmental behaviors like recycling or workplace pro-environmental behaviors require the contributions of many other fellow citizens to ensure their success. For instance, individuals are more likely to contribute to a public good if they observe that others also contribute [46, 89]. Estimation results reported in S9 Table suggests that social incentive factors play a significant role only when pro-environmental behaviors highly depend on the contribution of others.

## Discussions and conclusions

Our results suggest that policymakers should focus on at least three issues to promote pro-environmental behaviors in society. Firstly, we found that internal social influence is the most important social incentive that positively affects pro-environmental behaviors. This result means that internal social influence that motivates people to change their perceptions and attitudes is extremely important and necessary to promote pro-environmental behaviors. In addition to internal social influence, our results suggest that external social influence also positively impacts pro-environmental behaviors but is less effective than internal influences. This result aligns with the existing literature that holds that internal social motives are better than external ones because they guide people into changing their behaviors. In contrast, external influences can drive people to perform a specific action through compliance and identification, but it is not enough to motivate them to change their perceptions and attitudes toward a sustainable behavior [16, 17, 20]. This finding implies that the impacts of an environmental policy can be under-estimated if policymakers do not include social influence in their decisions regarding environmental issues. Therefore, based on our meta-analysis results, effective environmental policies should focus on strengthening individuals’ personal norms by fostering environmental awareness and the sense of obligation toward eco-friendly behaviors (e.g., improving green education and providing environmental information).

Secondly, since network is a valuable source of knowledge and information for individuals, the effectiveness of a conservation policy can be improved only if connections or interactions among individuals are increased. This result does not support an existing conjecture [90], which hypothesizes that increasing interactions between individuals in a large structure can be harmful to collective conservation behaviors. One example validating our results relates to pecuniary and non-pecuniary mechanisms in a spatial coordination game [91], consisting of giving agglomeration bonuses to people who interact in an enlarged network. It was shown that these bonuses could enhance coordination towards environmental conservation programs. In addition to the agglomeration bonus that encourages people to collaborate in a network to achieve an environmental target (i.e., collective actions), policymakers could also try to establish conditions under which individuals could share their knowledge to better drive them toward more sustainable behaviors (e.g., a favorable regulatory framework for environmental associations/groups).

Finally, we found that trust in institutions (e.g., governments, institutions or leaders) is needed to ensure a positive impact on pro-environmental behaviors. It is important because citizens’ trust in government can dictate individual behaviors to shape policy design and generate desired policy outcomes. For instance, trust in institutions could reduce the risk of free-riding and opportunistic behaviors as citizens would be willing to sacrifice some immediate personal benefits (e.g., by contributing to common goods) if they have positive expectations of the long-term outcomes of the government’s policies [92]. Examples of making institutions more inclusive, transparent, receptive and efficient at the local and national levels include increased transparency, improved communication and interaction with populations [83, 93] (e.g., participatory democracy and citizen convention).

This study quantified the strength and relevance of seven groups of social incentives of pro-environmental behaviors, which constitutes our main contribution to this study. One issue which has not been fully addressed is the impact of a country’s cultural differences on pro-environmental behaviors. Our meta-regression partially addressed this issue by using geographical regions and study-specific effects. The coefficient of MEA (i.e., Middle East & Africa) is positive and significant (although at the 10% significance level only), supporting a relatively higher effect compared to Europe (as the base category). However, we admit that our approach cannot satisfactorily address the differences in national cultures. This issue is important enough to be investigated in-depth in a future study, in which the general characteristic of a national culture can be captured by using Hofstede’s values, for example, and adopting a previously proven approach [94].

## Supporting information

S1 Checklist (DOC)

S1 Fig The diagram of these seven groups of social incentives. (PNG)

S2 Fig Plot of partial correlation coefficient vs. squared root of study’s sample size. (PDF)

S3 Fig Plot of partial correlation coefficient vs. publication year. The line and the shaded area represent the linear fit and the corresponding 95% confidence interval, respectively.
(PDF)

S1 Table Study characteristics. (TEX)

S2 Table Correlation matrix of seven social incentive dummies. (TEX)

S3 Table Definitions of dependent variable. (TEX)

S4 Table Definitions of predictor variables. (TEX)

S5 Table Descriptive statistics. (TEX)

S6 Table Test for publication bias. (TEX)

S7 Table Meta regression results. (TEX)

S8 Table Classifications of dependent variable. (TEX)

S9 Table Mixed-effect meta regression results with subgroups of dependent variable. (TEX)

S10 Table Summary survey table for meta-analysis of pro-environmental behaviors. (TEX)
