# Supplementary figures and images for: Social incentive factors in interventions promoting sustainable behaviors: A meta-analysis
Source: PLoS One. 2021 Dec 8;16(12):e0260932. doi: 10.1371/journal.pone.0260932 (PMC8654165; doi:10.1371/journal.pone.0260932)

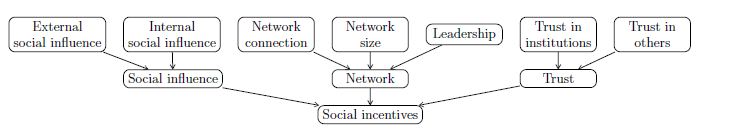

Supplement: S1 Fig — (PNG) [file pone.0260932.s002.PNG]

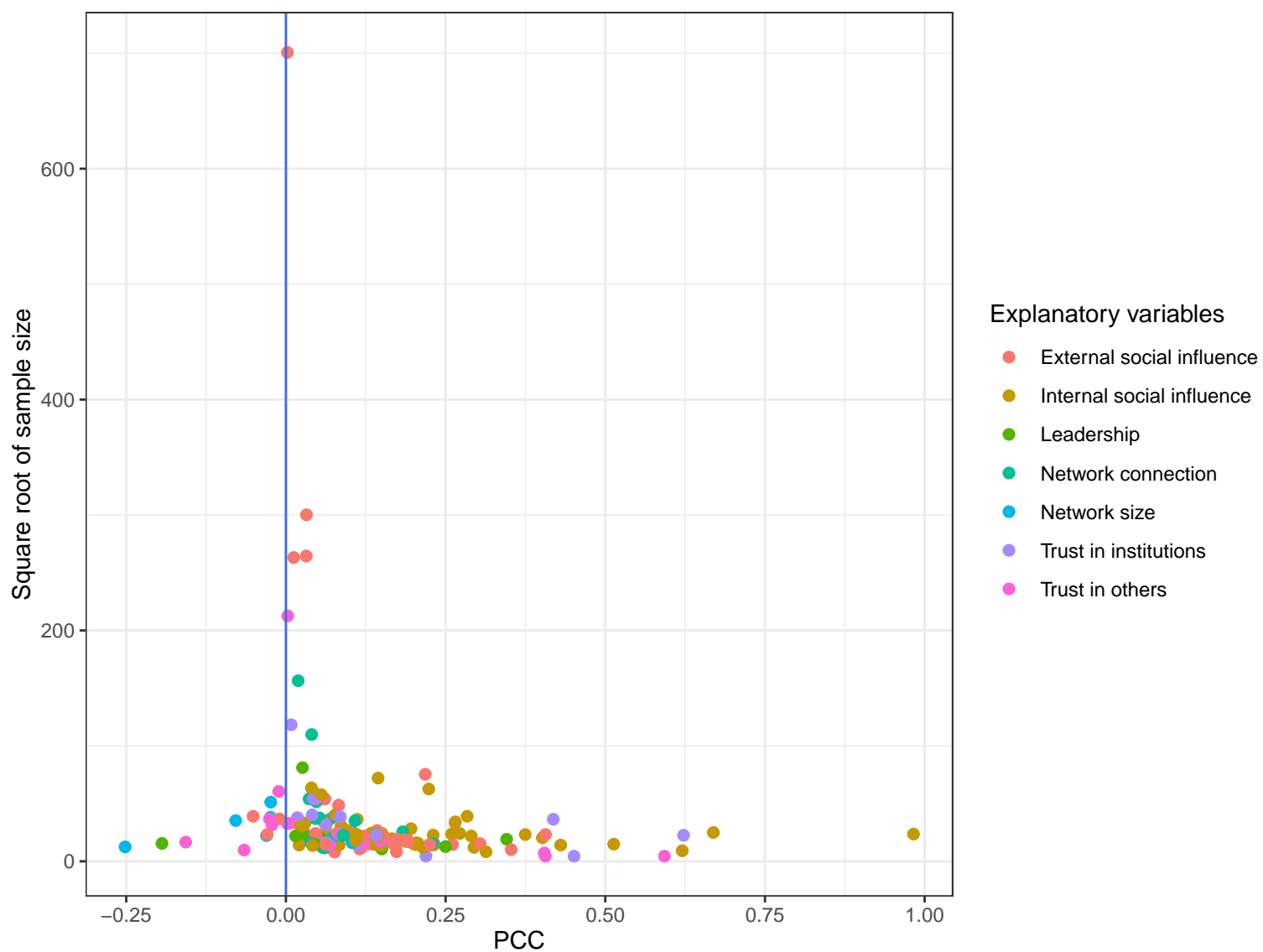

Supplement: S2 Fig — (PDF) [file pone.0260932.s003.pdf]

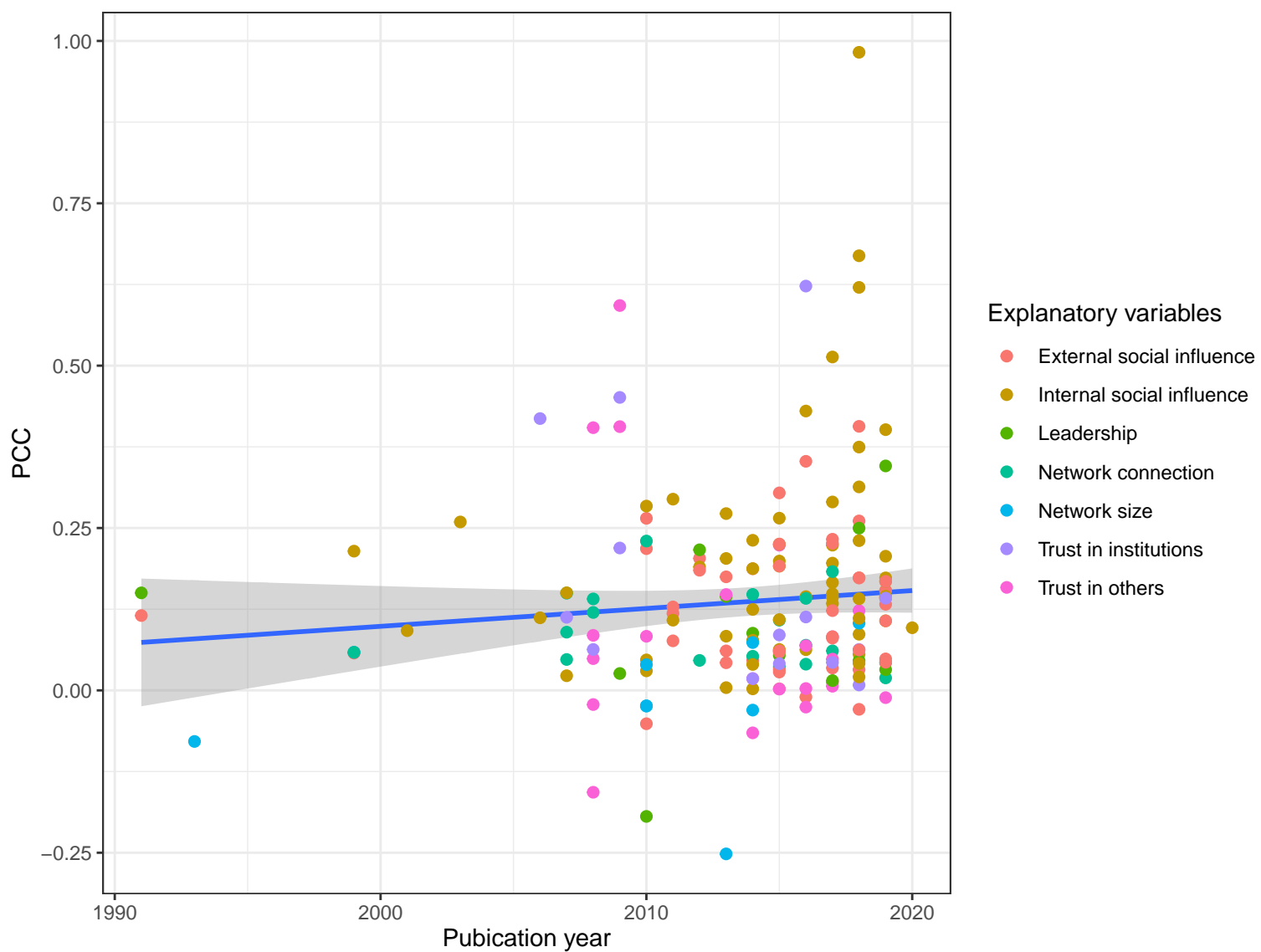

Supplement: S3 Fig — The line and the shaded area represent the linear fit and the corresponding 95% confidence interval, respectively. (PDF) [file pone.0260932.s004.pdf]
